# Frequent Engagement of RelB Activation Is Critical for Cell Survival in Multiple Myeloma

**DOI:** 10.1371/journal.pone.0059127

**Published:** 2013-03-28

**Authors:** Françoise Cormier, Hélène Monjanel, Claire Fabre, Katy Billot, Elène Sapharikas, Fanny Chereau, Didier Bordereaux, Thierry J. Molina, Hervé Avet-Loiseau, Véronique Baud

**Affiliations:** 1 INSERM, U1016, Institut Cochin, Paris, France; 2 CNRS, UMR8104, Paris, France; 3 Université Paris Descartes, Sorbonne Paris Cité, Paris, France; 4 Service d'hématologie et thérapie cellulaire, Centre Hospitalier Universitaire, Tours, France; 5 Département d'oncologie médicale, Institut Curie, Paris, France; 6 Département de Pathologie, Hôpital Hôtel-Dieu, Paris, France; 7 Laboratoire d'Hématologie, Centre Hospitalier Universitaire, Nantes, France; 8 Unité de Génomique du Myélome, Centre Hospitalier Universitaire, Toulouse, France; Westmead Millennium Institute, University of Sydney, Australia

## Abstract

The NF-κB family of transcription factors has emerged as a key player in the pathogenesis of multiple myeloma (MM). NF-κB is activated by at least two major signaling pathways. The classical pathway results in the activation of mainly RelA containing dimers, whereas the alternative pathway leads to the activation of RelB/p52 and RelB/p50 heterodimers. Activating mutations in regulators of the alternative pathway have been identified in 17% of MM patients. However, the status of RelB activation *per se* and its role in the regulation of cell survival in MM has not been investigated. Here, we reveal that 40% of newly diagnosed MM patients have a constitutive RelB DNA-binding activity in CD138^+^ tumor cells, and we show an association with increased expression of a subset of anti-apoptotic NF-κB target genes, such as cIAP2. Furthermore, we demonstrate that RelB exerts a crucial anti-apoptotic activity in MM cells. Our findings indicate that RelB activation is key for promoting MM cell survival through the upregulation of anti-apoptotic proteins. Altogether, our study provides the framework for the development of new molecules targeting RelB in the treatment of MM.

## Introduction

Multiple myeloma (MM) is a neoplastic plasma cell disorder that accounts for 1% of all cancers and more than 10% of all hematological malignancies [1]. MM is characterized by the clonal proliferation of plasma cells within the bone marrow microenvironment, associated with a monoclonal protein in the blood or urine, and organ dysfunction [2], [3]. Despite major therapeutic advances such as proteasome inhibition [4], MM remains an incurable disease, emphasizing the need for new targeted therapies. NF-κB has emerged as a crucial player in the pathogenesis of MM, particularly through the regulation of target genes involved in cell proliferation and survival [5]–[7]. Constitutive NF-κB activity is present in human MM cell lines and patient MM cells and associated with proteasome inhibitor sensitivity [8]–[10]. In the context of the bone marrow microenvironment, adhesion of MM cells induces NF-κB-dependent cytokine secretion, further enhancing NF-κB activity [8], [11], [12]. Therefore NF-κB has emerged as a promising therapeutic target in MM.

RelB belongs to the NF-κB family that consists of five members in mammals: RelA (p65), RelB, c-Rel, NF-κB1 (p50 and its precursor p105), and NF-κB2, (p52 and its precursor p100) [13], [14]. Analysis of RelB-deficient mice has shed light on the importance of RelB in B-cell maturation and secondary lymphoid organ development [15]–[17]. RelB^−/−^ mice also spontaneously develop a multiorgan inflammatory syndrome that contributes to premature mortality [15], preventing long-term studies on B-cell neoplasm development. There are indications suggesting that RelB can act as a negative regulator of cell survival in human diffuse large B-cell lymphoma cell lines with constitutive Malt1 activity [18], whereas its activation in non-Hodgkin's B lymphoma cells is associated with protection against apoptosis [19]. However, whether RelB is functionally important for the survival of MM cells has not been investigated.

The activity of RelB is in part regulated through the activation of the alternative or non-canonical NF-κB pathway which is stimulated by a restricted set of developmental cytokines such as lymphotoxinβ (LTβ), B-cell activating factor (BAFF) and CD40 ligand (CD40L) [8], [20], [21]. This pathway is dependent on NF-κB inducing kinase (NIK)-mediated activation of IKKα, thereby leading to phosphorylation and proteasome-dependent processing of p100, the main RelB inhibitor, and resulting in RelB/p50 and RelB/p52 nuclear translocation. Beyond the alternative NF-κB signaling cascade, RelB-dependent DNA binding activity is negatively regulated at the nuclear level by several mechanisms, such as trapping in RelA/RelB or p100/RelB complexes, and specific serine phosphorylation [22]–[26]. RelB containing dimers also display DNA binding specificity [27]–[29], and RelB recruitment to some genes correlates with transcriptional down-regulation (IL12-p40), whereas in other cases (EBV-induced molecule 1 ligand chemokine (ELC) and macrophage-derived chemokine (MDC)), it increases transcriptional activity well over the level achieved by RelA or cRel [30], further emphasizing the importance and unique role of RelB.

Multiple and recurrent genetic abnormalities in genes encoding regulators of the alternative NF-κB pathway (e.g. loss-of-function mutations in TRAF2/3 and cIAP1/2, gain-of-function mutations in NIK, and C-terminal truncation of p100) have been identified in MM cell lines and patient samples [31], [32], suggesting the involvement of the alternative NF-κB pathway in MM pathogenesis. However, whether RelB DNA-binding activity is constitutively activated in MM patient samples, and how it affects anti-apoptotic gene expression is not elucidated.

In the study presented here, we reveal a constitutive RelB DNA-binding activity in 21 out of 52 (approximately 40%) newly diagnosed MM patients, which is associated with high expression of a subset of anti-apoptotic NF-κB target genes, such as cIAP2. Furthermore, we demonstrate that RelB plays a crucial pro-survival role in MM cells, at least in part, *via* the transcriptional up-regulation of a subset of anti-apoptotic NF-κB target genes. Altogether, our data provide the framework for the development of new drugs targeting RelB to overcome chemoresistance in MM.

## Materials and Methods

### Ethics Statement

This study was approved by the ethics committee of the Centre Hospitalier Universitaire de Nantes where samples were collected. All samples were obtained with written informed consent reviewed by the ethical board of the same hospital as per the Declaration of Helsinki.

### Human MM cell lines

MM cell lines were obtained from the DSMZ collection (Braunschweig, Germany). Cells were grown in RPMI-1640 medium supplemented with 10–15% heat-inactivated fetal bovine serum (HyClone), 2 µM L-glutamine, 100 U/mL penicillin, and 100 µg/mL streptomycin (Invitrogen).

### Tumor cells from MM patients

Tumor cells from newly diagnosed MM patients were obtained from bone marrow aspirates after informed consent as per the Declaration of Helsinki. Mononuclear cells were separated using Ficoll Hypaque density sedimentation, and plasma cells were purified (>95% CD138^+^) by positive selection with anti-CD138 magnetic activation cell separation microbeads (StemCell Technologies, Vancouver, Canada).

### Antibodies

The antibodies were purchased from Santa Cruz (RelA, RelB, p105/p50, p100/p52, c-Rel), Sigma (β-actin) and Cell Signaling (cleaved caspase 3 (Asp175), Bcl-xL, Bcl-2, and cIAP2).

### Plasmids

pSuper vector containing polymerase III H1 promoter was provided by T. Tuschl (The Rockfeller University, New-York, USA). The lentiviral vectors pTrip-shRNA RelB and pTRIP-shRNA RelA were generated by subcloning an oligonucleotide designed to target RelB and RelA, respectively, under control of the H1 promoter into pTRIP-ΔU3-MND-GFP lentiviral vector [33]. pTRIP-shRNA control was generated following the same approach using a scrambled control oligonucleotide. RelB sense 5′-GATCCCCGGAGATCATCGACGAGTACTTCAAGAGAGTACTCGTCGATGATCTCCTTTTTGAATTCA-3′; RelA sense 5′-GATCCCCGCATCCAGACCAACAACAATTCAAGAGATTGTTGTTGGTCTGGATGCTTTTTGAATTCA-3′; control sense 5′-GATCCCCCGTACGCGGAATACTTCGATTCAAGAGATCGAAGTATTCCGCGTACGTTTTTGAATTCA-3′.

### Immunoblotting

Immunoblotting were performed as previously described [23].

### Electrophoretic mobility shift assay for NF-κB

NF-κB activation was analyzed by electrophoretic mobility shift assay (EMSA) using the human immunodeficiency virus long terminal repeat tandem κB oligonucleotide as κB probe as previously described [23]. For supershift assays, cell extracts were incubated with specific antibodies for 30 min on ice before incubation with the labeled probe.

### Lentiviral production and transduction

Production of infectious recombinant lentiviruses was performed by transient transfection of 293T cells as previously described [33]. For infections, cells were incubated overnight with recombinant lentiviruses. An equal amount of fresh culture medium was added, and an additional 24 h later, cells were washed and seeded in fresh culture medium. GFP positive cells were sorted with FACSAria III sorter (Becton Dickinson).

### siRNA transfections

siRNA transfections were performed as previously described [34]. siRNA duplex oligonucleotides targeting cIAP2 were synthesized by Sigma. cIAP2 sense: 5′- AACAGUGGAUAUUUCCGUGGC-dTdT-3′; scrambled control sense: 5′-CGUACGCGGAAUACUUCGAdTdT-3′.

### Annexin V binding assay

Cells were harvested and Annexin V binding was performed according to the manufacturer's instructions (Becton Dickinson). Cells were subjected to cytometric analysis with a FACSCalibur cytometer (Becton Dickinson) by using the CellQuestPro software (Becton Dickinson).

### Measurement of mitochondrial transmembrane potential (Δψm)

For determination of mitochondrial transmembrane potential, cells were incubated with 100 nM Tetramethyl Rhodamine Methyl Ester (TMRM) (Molecular Probes) in PBS for 15 min at room temperature. Cells were subjected to cytometric analysis as above.

### RT-qPCR

Total RNA extraction and reverse transcription were performed as previously described [23]. Real-time PCR analysis was carried out with LightCycler FastStart DNA Master plus SYBR Green I on a Light Cycler 1.5 (Roche Applied Science). All values were normalized to the level of HPRT mRNA. Primer sequences are as follow: cIAP2 sense 5′-ACTAATACCGGGAACA-3′; cIAP2 antisense 5′-ACTCCTGGGCTCAAGTAATTC-3′; Bcl-xL sense 5′-CCCGACCTGTGATACAA-3′; Bcl-xL antisense 5′-ATCCAAAGCCAAGATAAGATT-3′; Bcl-2 sense 5′-GTCTGGGAATCGATCTGGAA-3′; Bcl-2 antisense 5′-GCAACGATCCCATCAATCTT-3′; XIAP sense 5′-GCAAGAGCTCAAGGAGACCA-3′; XIAP antisense 5′- AAGGGTATTAGGATGGGAGTTCA-3′; TRAF2 sense 5′- GCATACCCGCCATCTTCTC-3′; TRAF2 antisense 5′- CGTTCAGGTAGATACGCAGACA-3′; RelA sense 5′-TTGAGCCCACAAAGCCTTATCAAGT-3′; RelA antisense 5′-GGACAATGCCAGTGCCATACAG-3′; RelB sense 5′-CTCACTCTCGCTCGCCGTTTC-3′; RelB antisense 5′-CACAGGGCCCAGGGTGACCGT-3′; HPRT sense 5′-GGCGTCGTGATTAGTGATG-3′; HPRT antisense 5′-GCACACAGAGGGCTACAATGT-3′.

### Chromatin immunoprecipitation (ChIP) assays

ChIP assays were performed as described [34]. Samples were analyzed by real-time PCR. Sequences of promoter-specific primers are available upon request.

### Statistical analysis

Statistical significance was assessed using unpaired t tests (Prism 5.0c, GraphPad Software). A value of *P<0.05* was considered as statistically significant with the following degrees: *P<0.05; **P<0.01; ***P<0.001.

## Results

### RelB is constitutively activated in primary CD138^+^ cells from newly diagnosed MM patients

**Figure 1:**
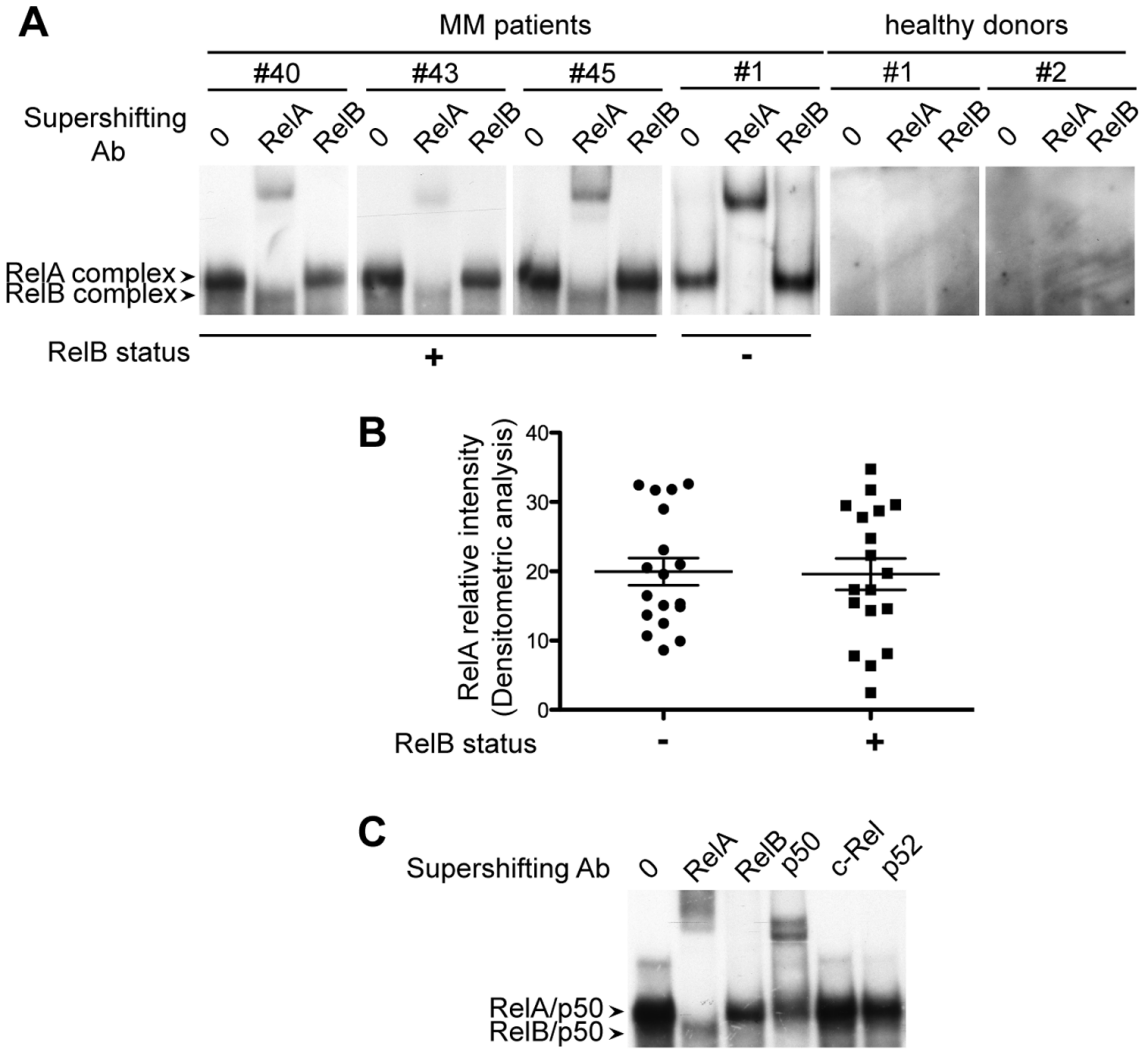
RelB is constitutively activated in primary CD138^+^ cells from newly diagnosed MM patients. (A) Whole cell extracts from primary CD138^+^ cells isolated from 52 newly diagnosed MM patients and two healthy donors were analyzed for NF-κB activity by EMSA. Four representative MM cases are presented (see Table 1 for tabulated study report). For supershift, whole cell extracts were either left untreated or incubated with either anti-RelA or anti-RelB antibodies for 30 min prior to incubation with the labeled probe. RelA- and RelB-containing complexes are indicated. (B) RelB binding activity is not associated with significant differences in the intensity of RelA DNA-binding activity in MM patient samples. EMSA and supershift analyses were performed from 18 RelB-positive and 18 RelB-negative *de novo* MM patient samples as in (A). Relative signal intensity of RelA DNA binding activity normalized to a reference patient sample loaded on each gel (means ± SD of two independent experiments for each MM sample) is shown. (C) Subunit composition of RelA- and RelB-containing complexes. Whole cell extracts used in (A) were incubated with the indicated antibodies prior to incubation with the labeled probe. Data of one representative RelB-positive MM patient is shown (#45).

To directly assess the status of RelB activation in MM patients, we performed EMSA combined with supershift analysis using anti-RelB antibody to evaluate RelB DNA-binding activity in 52 purified MM samples from newly diagnosed patients, and 2 purified healthy donors. A constitutive binding of RelB was observed in 21 MM patients (40%) (Figure 1A, cases # 40, #43 and # 45; and Table 1 for tabulated study report). We also analyzed RelA DNA-binding activity in the same 52 MM patient samples, and observed that 39 MM patients (75%) are positive for RelA (Figure 1A, cases #40, #43, # 45 and #1; and Table 1). In contrast, neither RelA nor RelB activation was observed in plasma cells isolated from the 2 healthy donors (Figure 1A). Interestingly, although RelB-positive MM patient samples were all positive for RelA (Table 1), no significant differences on the intensity of RelA DNA-binding activity were seen between the RelB-positive- and RelB-negative-MM patient subgroups (Figures 1B). In a second step, to further determine the subunit composition of the RelA- and RelB-DNA complexes in RelB-positive MM patients, we performed a supershift analysis using antibody directed against each of the five subunits of the NF-κB family i.e. RelA, RelB, p50, p52 and c-Rel (Figure 1C). Antibody to p50 supershifted RelB complexes almost completely, together with a fraction of RelA complexes. Antibodies to p52 and c-Rel had very little effect on either complex. Altogether, our data demonstrate the frequent constitutive activation of RelB (approximately 40%) in primary CD138^+^ cells from newly diagnosed MM patients, irrespectively of the level of RelA activation.

**Table 1:** RelA- and RelB-DNA binding activity in CD138^+^ cells from newly diagnosed multiple myeloma patients.

Patient no.	Patient age y/sex	ISS	t(4;14)	del(17p)	RelA binding activity	RelB binding activity
1	64/F	2	Nd	0	+	−
2	72/M	1	0	0	+	+
3	61/M	3	0	0	+	+
4	49/F	1	0	0	+	−
5	63/F	3	0	0	−	−
6	72/M	2	0	0	+	−
7	65/M	3	1	0	+	−
8	76/F	1	0	0	+	+
9	65/M	2	0	0	+	+
10	66/M	2	nd	0	+	−
11	75/F	3	nd	nd	+	−
12	66/F	2	0	0	+	+
13	57/F	3	0	0	+	−
14	46/M	?	0	0	+	+
15	75/M	1	0	0	+	−
16	81/F	3	0	0	−	−
17	64/M	3	0	0	+	+
18	79/M	2	0	0	+	+
19	68/M	2	0	0	−	−
20	64/M	3	0	0	+	+
21	39/M	3	1	0	−	−
22	64/F	3	0	0	+	+
23	63/F	2	0	0	+	−
24	75/M	2	0	0	−	−
25	70/M	2	0	0	+	+
26	80/M	3	0	0	+	+
27	55/F	1	0	0	+	−
28	???				−	−
29	80/M	3	0	0	−	−
30	77/M	2	0	0	+	−
31	71/F	2	0	0	+	+
32	78/M	3	0	0	−	−
33	76/F	2	0	0	+	+
34	69/F	2	0	0	+	−
35	71/F	3	nd	nd	+	+
36	76/F	3	0	0	+	−
37	68/F	1	0	0	−	−
38	62/F	1	0	0	+	+
39	74/M	3	0	0	−	−
40	65/M	1	0	1	+	+
41	68/M	2	0	0	+	−
42	64/F	1	0	0	+	−
43	61/M	2	1	0	+	+
44	75/M	3	0	0	+	−
45	61/F	3	0	0	+	+
46	66/M	2	1	0	+	−
47	65/M	3	0	0	+	−
48	78/M	2	0	0	−	−
49	74/M	1	0	0	+	+
50	59/M	1	0	0	−	−
51	69/F	2	1	0	−	−
52	50/M	2	0	0	+	+

RelA- and RelB-DNA binding activity in CD138^+^ plasma cells from 52 newly diagnosed MM patients was evaluated by EMSA combined with supershift analysis. ISS indicates disease stage according to the International Staging System. Patients with t(4;14) or del(17p) genetic alterations are indicated.

### RelB constitutive activation is associated with up-regulation of anti-apoptotic NF-κB target gene expression in primary CD138^+^ cells from MM patients

**Figure 2:**
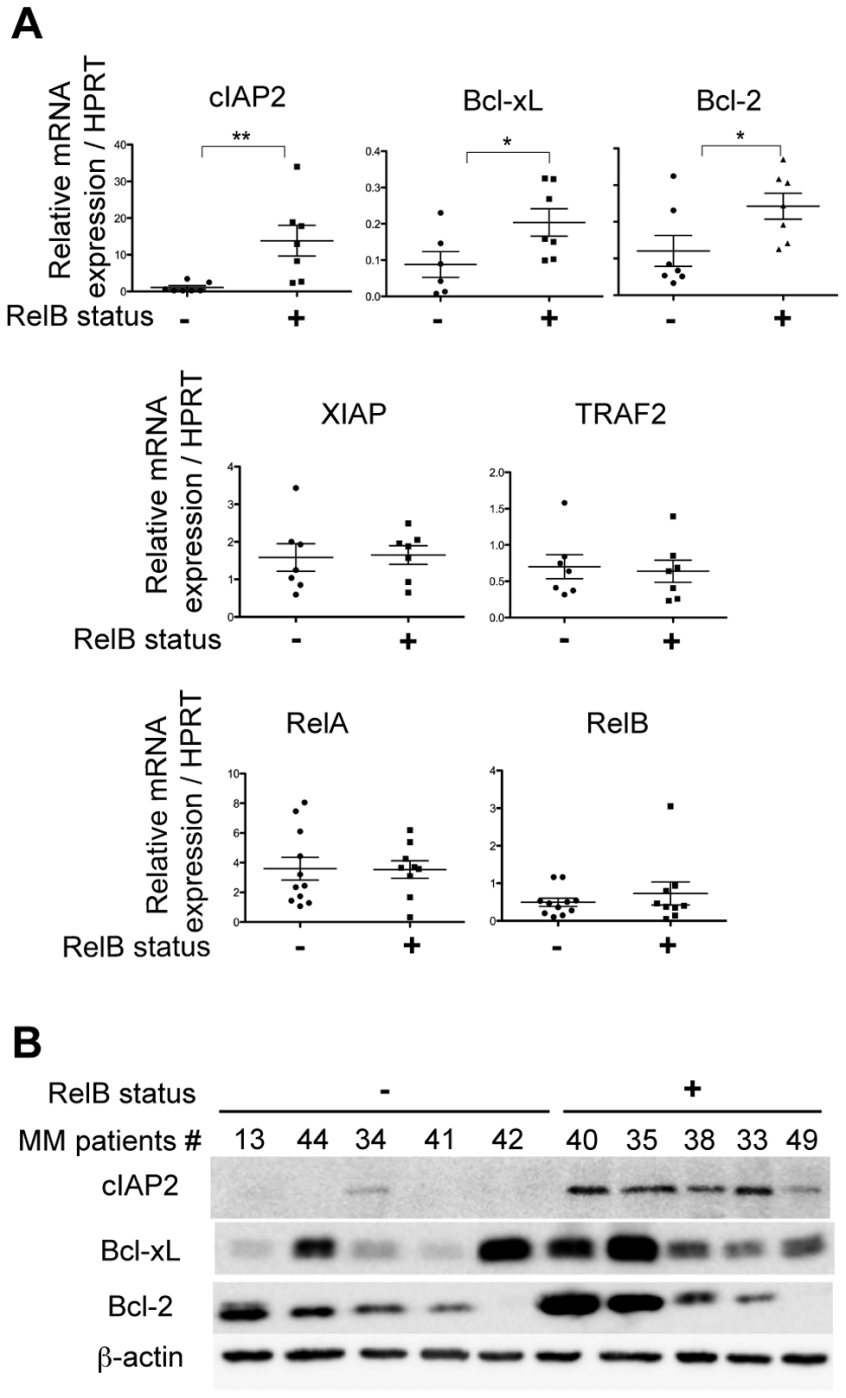
RelB constitutive activation is associated with increased expression of a subset of anti-apoptotic NF-κB target genes in primary CD138^+^ cells from MM patients. (A) RelB constitutive activation in CD138^+^ cell from MM patients is associated with increased anti-apoptotic NF-κB target gene mRNA expression. Quantitative RT-PCR was performed with specific primer pairs for the indicated genes using total RNAs prepared from 14 *de novo* MM patients either with or without RelB constitutive activation. Results are means ± SD of two independent experiments for each MM sample normalized to the level of HPRT mRNA. (B) RelB constitutive activation in CD138^+^ cells from MM patients is associated with increased anti-apoptotic NF-κB target gene protein expression. Whole cell extracts from primary CD138^+^ cells from MM patients tested in (A) were analyzed by immunoblotting for the indicated proteins. *P<0.05, **P<0.01.

It was important to determine whether RelB constitutive activation in MM cases could be associated with increased expression of endogenous NF-κB target genes. Therefore, using quantitative RT-PCR, we first compared mRNA levels of 15 known NF-κB target genes associated with cell proliferation (Cyclin D1, Cyclin E, IL-6, IL-1 and TNFα) and suppression of apoptosis (cIAP1, cIAP2, XIAP, survivin, A20, FasL, Bcl-xL, Bcl-2, TRAF1 and TRAF2) in RelB-positive- and RelB-negative-MM patient samples. Figure 2A presents the results obtained for 5 representative anti-apoptotic genes, cIAP2, Bcl-xL, Bcl-2, XIAP and TRAF2, that are differently expressed depending on the status of RelB activation. RelB constitutive activation is associated with a marked and significant increase in the expression of cIAP2 (P = 0.009), and to a lesser extent Bcl-xL and Bcl-2 (P = 0.048, and P = 0.047, respectively) (Figure 2A, upper panels). In contrast, XIAP and TRAF2 mRNA levels were not significantly dependent on the status of RelB activation (Figure 2A, middle panels). As a control, no significant effects on RelA and RelB expression were seen (Figure 2A, lower panels). The increase in mRNA levels of cIAP2, Bcl-xL and Bcl-2 was further confirmed at the protein level by a marked accumulation of cIAP2 protein in patients with a constitutive activation of RelB, and to a lesser extent Bcl-xL and Bcl-2 proteins (Figure 2B). Taken together, these results indicate that constitutive activation of RelB in MM patients is associated with increased expression of a subset of anti-apoptotic NF-κB target genes.

### RelB promotes anti-apoptotic NF-κB target gene expression in MM cell lines *via* direct transcriptional activation

**Figure 3:**
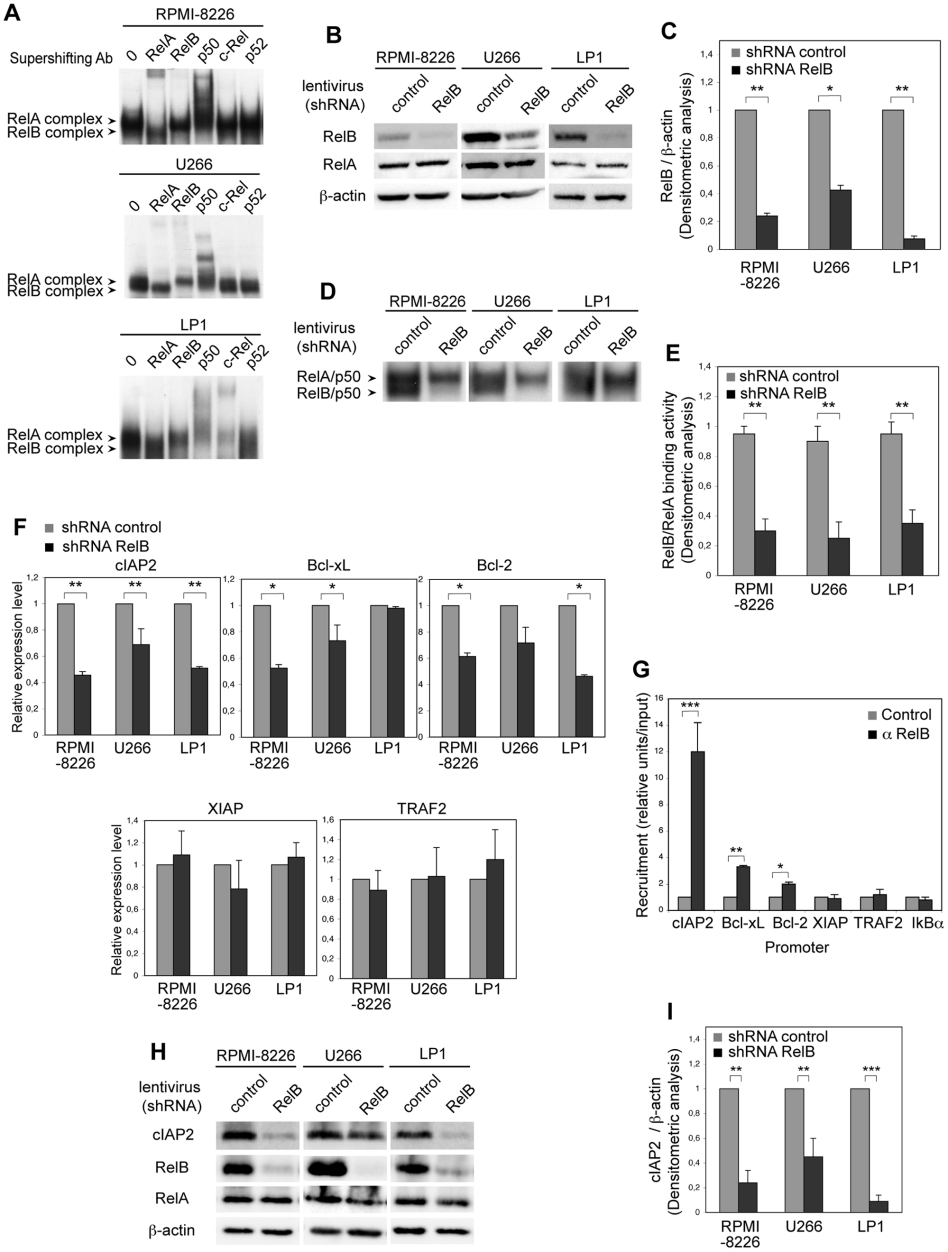
RelB promotes anti-apoptotic NF-κB target gene expression in MM cell lines *via* direct transcriptional activation. (A) RelB constitutive activation in MM cell lines. Whole cell extracts from RPMI-8226, U266, and LP1 cell lines were analyzed for NF-κB activity by EMSA. For supershift, whole cell extracts were incubated with the indicated antibodies prior to incubation with the labeled probe. RelA- and RelB-containing complexes are indicated. (B, C) RelB protein levels are efficiently knocked down by stable RNA interference. Whole cell extracts from RPMI-8226, U266, and LP1 cell lines transduced with lentiviruses encoding either a shRNA targeting RelB (shRNA RelB) or a scrambled control (shRNA control) were analyzed by immunoblotting for the indicated proteins. Data of a representative experiment are shown (B), and densitometric analysis of RelB protein level normalized to β-actin (means ± SEM of three independent experiments for each MM cell line) is reported (C). (D, E) RelB constitutive DNA-binding activity is efficiently knocked down by stable RNA interference. Whole cell extracts prepared as in (B) were analyzed by EMSA. RelA- and RelB-containing complexes are indicated. Data of one representative experiment are shown (D), and densitometric analysis of RelB DNA-binding activity normalized to RelA DNA-binding activity (means ± SEM of three experiments for each MM cell line) is reported (E). (F) RelB knockdown decreases anti-apoptotic NF-κB target gene expression at the mRNA level in MM cell lines. Quantitative RT-PCR was performed with specific primer pairs for the indicated genes using total RNAs prepared from the three MM cell lines upon RelB knockdown as in (B). Results are means ± SEM of three independent experiments for each MM cell line normalized to the level of HPRT mRNA. (G) RelB is constitutively bound to the *cIAP2*, *Bcl-xL* and *Bcl-2* promoters in RPMI-8226 MM cells. Recruitment of RelB to the *cIAP2*, *Bcl-xL*, *Bcl-2*, *TRAF2*, *XIAP* and *IκBα* promoters was examined by ChIP experiments followed by quantitative PCR analysis. Results are means ± SEM of four independent experiments normalized to inputs that reflect relative amount of sonicated DNA fragments present before immunoprecipitation, (H, I) RelB knockdown decreases cIAP2 protein expression levels in MM cell lines. Whole cell extracts prepared as in (B) were analyzed by immunoblotting for the indicated proteins. Data of one representative experiment are shown (H), and densitometric analysis of cIAP2 protein level normalized to β-actin (means ± SEM of three independent experiments for each MM cell line) is reported (I). ***P<0.001, **P<0.01, *P<0.05.

To directly assess the contribution of RelB on anti-apoptotic NF-κB signature expression in MM cells, we developed a stable RelB knockdown approach by RNA interference in RelB-positive MM cell lines using either a lentivirus carrying a shRNA targeting RelB or a scrambled control, and analyzed anti-apoptotic NF-κB target gene expression. We first confirmed, as previously described [35], [36], that RPMI-8226 and U266 exhibit a strong constitutive RelB DNA-binding along with RelA DNA-binding activity (Figure 3A, upper and middle panels). In addition, we identified LP1 as presenting a constitutive activation of RelB (Figure 3A, lower panel). Of note, RPMI-8226, U266 and LP1 cell lines carry a TRAF3 inactivating mutation that represents the most common abnormality identified in the alternative NF-κB pathway [37]. As shown in Figures 3B and 3C, RelB protein levels were efficiently and significantly decreased in RPMI-8226, U266 and LP1 cells infected with a lentivirus carrying a shRNA directed against RelB compared to what is seen in the empty-virus infected cells. Importantly, RelB knockdown by RNA interference also resulted in a marked decrease in constitutive RelB binding activity without affecting RelA binding activity (Figures 3D and 3E). Knockdown of RelB expression in RPMI-8226, U266 and LP1 cells markedly and significantly decreased cIAP2 mRNA levels (Figure 3F, upper panels, *left*, P = 0.0017, P = 0.0034, and P = 0.0014, in RPMI-8226, U266 and LP1, respectively), and to a lesser extent Bcl-xL (Figure 3F, upper panels, *center*, P = 0.028 and P = 0.048 in RPMI-8226 and U266, respectively), and Bcl-2 (Figure 3F, upper panels, *right*, P = 0.021 and P = 0.018 in RPMI-8226 and LP1, respectively). In contrast, no differences in mRNA levels of XIAP and TRAF2 were observed upon RelB knockdown (Figure 3F, lower panels). Further *in vivo* evidence for a specific role of RelB on *cIAP2*, *Bcl-xL* and *Bcl-2* transcription was obtained by chromatin immunoprecipitation (ChIP) analysis. As shown in Figure 3G, RelB is constitutively recruited to the *cIAP2* (P = 0,0006), *Bcl-xL* (P = 0,0069) and *Bcl-2* (P = 0,0166) promoters. As a control, RelB was not seen to bind to the *TRAF2*, *XIAP* and *IκBα* promoters. In concert with these results, the amount of cIAP2 protein was markedly and significantly decreased in RPMI-8226, U266 and LP1 cells infected with a lentivirus carrying a shRNA directed against RelB compared to what is seen in the empty-virus-infected cells (Figures 3H and 3I), thus strengthening the observations made in MM patient samples (Figures 2A and 2B). Taken together, these results indicate that RelB upregulates the expression of a subset of anti-apoptotic NF-κB target genes in MM cell lines and patient samples *via* a direct transcriptional control.

### RelB constitutive activation is critical for MM cell survival

**Figure 4:**
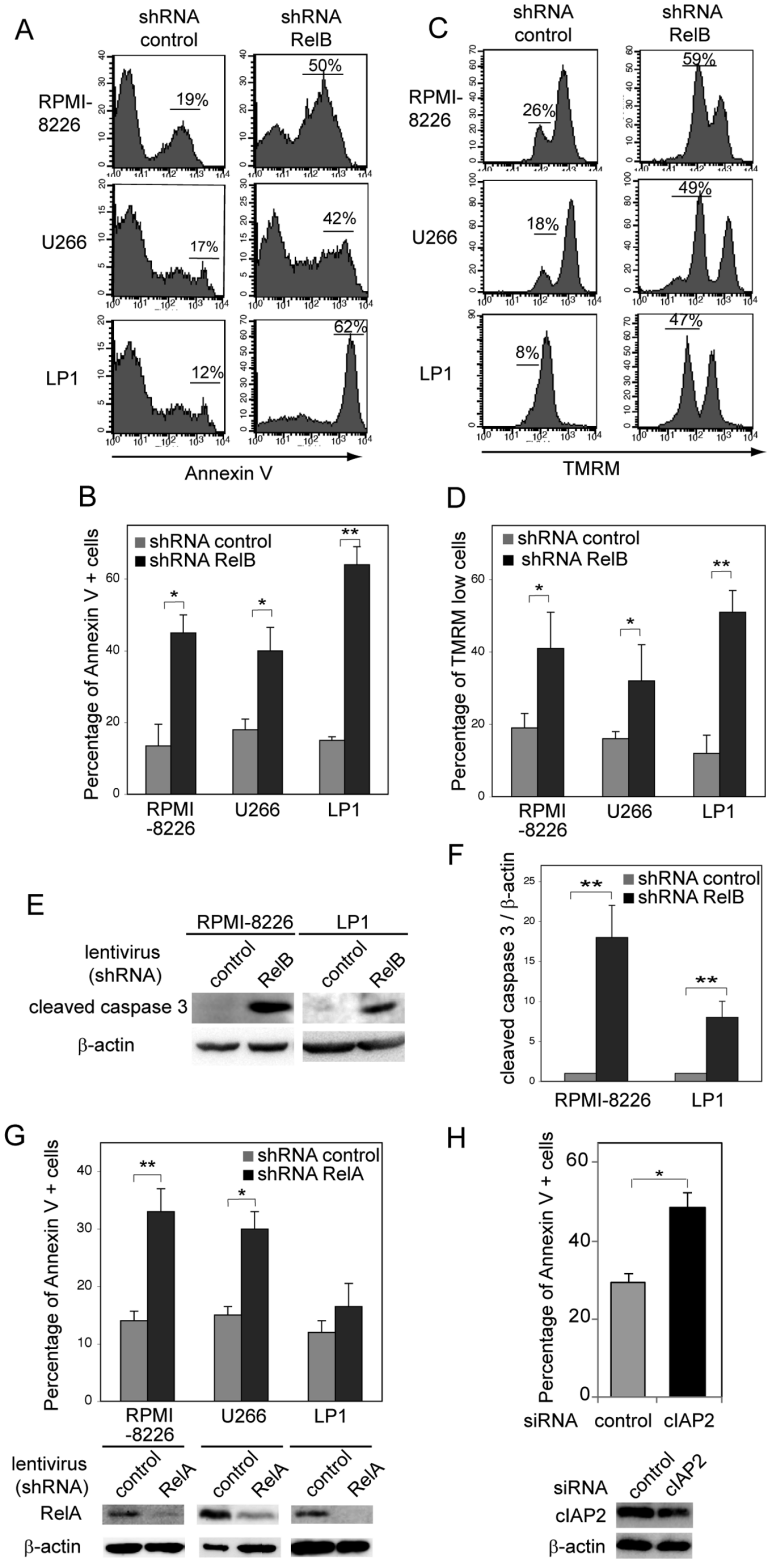
RelB is critical for MM cell survival. (A–D) RelB knockdown induces MM cell apoptosis. RPMI-8226, U266, and LP1 cell lines transduced with lentiviruses encoding either a shRNA targeting RelB or a scrambled control were monitored for apoptosis by annexin V-PE staining followed by FACS analysis. Data of one representative experiment are shown (A), and means ± SEM of three independent experiments for each MM cell line is reported (B). (C, D) RelB knockdown induces loss of mitochondrial transmembrane potential in MM cells. Loss of mitochondrial transmembrane potential was monitored by TMRM staining followed by FACS analysis. Data of one representative experiment are shown (C), and means ± SEM of three independent experiments for each MM cell line is reported (D). (E, F) RelB knockdown induces caspase 3 cleavage in MM cells. Whole cell extracts from RPMI-8226 and LP1 cell lines transduced with lentiviruses encoding either a shRNA targeting RelB or a scrambled control were analyzed by immunoblotting for the indicated proteins. Data of one representative experiment are shown (E), and densitometric analysis of cleaved caspase 3 protein levels normalized to β-actin (means ± SEM of two independent experiments for each MM cell line) is reported (F). (G) RelA knockdown induces apoptosis in RelB-positive MM cell lines. RPMI-8226, U266, and LP1 cell lines transduced with lentiviruses encoding either a shRNA targeting RelA or a scrambled control were monitored for apoptosis by annexin V-PE staining followed by FACS analysis. Means ± SEM of three independent experiments for each MM cell line is reported. Efficient knockdown of RelA protein levels was analyzed by immunoblotting (lower panels) (H) cIAP2 knockdown by RNAi induces MM cell apoptosis. LP1 cells were transfected with either a scrambled control sequence (siRNA control) or a siRNA oligonucleotide targeting cIAP2. Six days after transfection, cell apoptosis was monitored as in (A). Means ± SEM of three independent experiments is reported. Efficient knockdown of cIAP2 protein levels was analyzed by immunoblotting (lower panels) 3 days post-transfection. *P<0.05, **P<0.01.

Given that RelB upregulates the expression of anti-apoptotic NF-κB responsive genes, we asked whether modulation of RelB level might also affect MM cell survival. Knockdown of RelB expression in RPMI-8226, U266 and LP1 cells induced a marked apoptosis as evaluated by increase in annexinV-positive cells (Figures 4A and 4B, mean: 13,5% *versus* 45% in RPMI-8226, 18% *versus* 40% in U266, and 15% *versus* 64% in LP1), increase in loss of mitochondrial transmembrane potential Δψm (Figures 4C and 4D, mean: 19% *versus* 41% in RPMI-8226, 16% *versus* 32% in U266, and 12% *versus* 51% in LP1), and induction of caspase 3 cleavage (Figures 4E and 4F). Next, all RelB-positive MM samples and cell lines being also positive for RelA, we wanted to see the contribution of RelA in RelB-positive MM cell survival. We developed a stable RelA knockdown approach by RNA interference in MM cell lines, and monitored apoptosis. As shown in Figures 4G, RelA protein levels were efficiently repressed in RPMI-8226, U266 and LP1 cells infected with a lentivirus carrying a shRNA directed against RelA compared to what is seen in the empty-virus infected cells; and apoptosis was markedly increased in RPMI-8226 and U266 cells, and to a lesser extent in LP1 cells (mean: 14% *versus* 33% in RPMI-8226, 15% *versus* 31% in U266, 12% *versus* 18% in LP1), thus indicating that overall NF-κB activation, including both RelA and RelB, is critical for MM cell survival. Further, since cIAP2 emerged as an anti-apoptotic NF-κB target gene most notably regulated by RelB in RelB-positive MM cell lines, we examined the direct contribution of cIAP2 on MM cell survival. As shown in Figure 4H, apoptosis of LP1 cells was significantly increased upon cIAP2 expression knockdown compared with that seen with the scrambled control siRNA (mean 29% *versus* 49%, P = 0,022). Collectively, our data indicate that RelB exerts a crucial anti-apoptotic activity in MM cells by upregulating the expression of a subset of anti-apoptotic NF-κB target genes, such as cIAP2.

## Discussion

Aberrant NF-κB activity has been observed in many cancers, including both solid and hematopoietic malignancies, and sustained activation of NF-κB can affect all six hallmarks of cancer [38], including insensitivity to growth inhibitory signals, evasion of apoptosis, induction of angiogenesis, and metastasis [8], [39]–[41], thus leading to the concept of NF-κB addiction in cancer cells. However, although the regulation of specific target genes by individual NF-κB subunits has emerged as an important mechanism to achieve the required specificity and selectivity of the NF-κB response [29], [42], [43], the precise contribution of individual NF-κB subunits in MM pathogenesis has not been characterized.

In the present study, we conducted the first extensive analysis of RelB DNA-binding activity on a large cohort of 52 newly diagnosed MM patients. Our data emphasize that constitutive activation of RelB is frequent (approximately 40%) in MM cases, indicating that it represents a major event in MM. Moreover, we uncovered a crucial role for RelB in promoting MM cell survival *via* the increased expression of a subset of anti-apoptotic NF-κB target genes by a direct transcriptional control. These results have important implications for the role of RelB in MM pathogenesis and therapy.

The alternative NF-κB pathway is dependent on NIK-mediated activation of IKKα, thus leading to the phosphorylation and proteasome-dependent processing of cytoplasmic p100, and resulting in RelB/p52 and RelB/p50 nuclear translocation [8], [20], [21]. Two reports have highlighted the relationship between NF-κB gene expression signature and genetic abnormalities in regulators of the alternative NF-κB signaling cascades in 9% [32] and 17% [31] of patient cohorts with MM, suggesting the biological significance of the alternative NF-κB pathway in MM pathogenesis. However, RelB activation *per se* and its physiological significance have not been investigated. Here, we demonstrate that RelB is constitutively activated in approximately 40% of MM cases, a much higher frequency than expected based on evaluation of the alternative NF-κB signaling cascade activation by cancer genomic approaches. One can hypothesized that important regulators of the alternative NF-κB pathway remain to be identified, and thereby their putative mutations being overlooked. Alternatively, additional mechanisms might contribute to the regulation of RelB activity in MM cells. This hypothesis is supported by the observation that knockdown of IKKα, the physiological target of NIK, has no effect on RelB binding activity in MM cells [36]. First, Fbxw7α and GSK3-mediated proteosomal degradation of nuclear p100 has recently emerged to account for the control of RelB transcriptional activity and MM cell survival [44]. Second, because the MM cell line RPMI 8226 harbors constitutive RelB activity maintained by the recently described proteasome inhibitor-resistant (PIR) pathway [35], it is possible that RelB activation is induced by such atypical proteasome-independent mechanisms in malignant plasma B cells isolated from newly diagnosed MM patients. Third, NF-κB is frequently activated in malignant cells, particularly MM cells, in response to inflammatory stimuli originating from the microenvironment, and not because of intrinsic mutations [45]. The adherence of MM cells to bone-marrow stromal cells (BMSCs) induces NF-κB-dependent cytokine transcription and secretion (e.g. IL-6, TNFα and BAFF) by BMSCs, which in turn promote MM cell growth and survival through paracrine mechanisms [2], [11], [46]. Although both classical and alternative pathways are activated by coculture of MM cells with BMSCs [36], the relative contribution of the microenvironment in mediating RelB activation in MM tumor cells is poorly understood and worth further investigation. Finally, EMSA experiments have shown that RelB activation in MM cases is not significantly associated with variations in the level of RelA DNA binding (Figure 1B), suggesting that RelB exerts its activity irrespectively of RelA. Nonetheless, it is important to note that RelB activation does not make RelA less critical for the survival of MM cells. Indeed, we have observed that RelB-positive patient samples were also positive for RelA, and knockdown of RelA expression in MM cell lines leads to a marked increase in apoptosis, thus supporting the idea that an efficient anti-NF-κB therapeutic approach should target both RelA and RelB activation in MM.

Our study has revealed that RelB promotes tumor cell survival in MM. In addition, we have demonstrated that RelB activity is required for optimal expression of a subset of anti-apoptotic NF-κB target genes in MM cells, most strikingly cIAP2, and ChIP experiments have shown that RelB is recruited to the region that encompasses the NF-κB binding sequence of the promoter of these anti-apoptotic genes. Altogether, we propose that the pro-survival activity of RelB in MM cells is exerted through κB-site and DNA-binding-dependent recruitment of RelB to anti-apoptotic NF-κB target genes. In support to this hypothesis, we have demonstrated that cIAP2 expression plays a crucial anti-apoptotic function in RelB-positive MM cell lines. Interestingly, cIAP2 belongs to the NF-κB gene signature reported as a marker of NF-κB activation in MM cell lines and patient samples [32], [37]. Thus, our results strongly suggest that increased cIAP2 expression is directly controlled by RelB heterodimers in MM tumor cells. Although cIAP2 was originally characterized by its ability to inhibit cell death through suppression of caspase activity [47], it has more recently emerged that cIAP2 is also involved in many aspects of innate and adaptive immune cell function through the ubiquitin-mediated regulation of the NF-κB pathways [48], [49]. The role of cIAP1/2 in NF-κB regulation and function is highly complex, and is dependent of the cellular context. In normal cells, cIAP2 acts as a negative regulator of the alternative NF-κB pathway through NIK proteosomal degradation *via* its E3 ligase activity. However, in the pathological context of MM cells, it has been proposed that any mutations-which are frequent in MM cells- affecting the binding of cIAP2 to other crucial NF-κB regulators, especially cIAP1, TRAF2/3 and NIK, would result in NIK stabilization. Overexpression of NIK in turn leads to constitutive activation of the classical and alternative pathways, and an increase in B-cell growth and survival. In regards to our results, we propose that RelB-dependent increase in cIAP2 expression constitutes a strong pro-survival signal in MM cells through its anti-apoptotic activity. Beyond MM, close relation between anti-apoptotic gene expression and RelB activity has emerged in other B-cell neoplasms. Inhibition of Notch-induced RelB/p52 activity in Hodgkin lymphoma cell lines is associated with apoptosis and decreased expression of cIAP2 [50]. Moreover, BMSCs prevent apoptosis of primary B lymphoma cells, at least in part, through RelB-dependent increased expression of NF-κB-dependent anti-apoptotic genes (including cIAP1/2 and XIAP) [19]. Thus, it is likely that the prosurvival effects of RelB observed in MM may be generalized to other B-cell neoplasms, especially those addicted to NF-κB.

In conclusion, we established that RelB is a crucial positive regulator of MM cell survival frequently activated in MM patient samples. Our data are of great functional importance because they constitute a significant advance in the understanding of RelB physiological function and provide a strong rationale for the development of new molecules targeting RelB in the treatment of MM.
